# Spillover and crossover effects of working time demands on work–life balance satisfaction among dual-earner couples: the mediating role of work–life conflict

**DOI:** 10.1007/s12144-022-03850-0

**Published:** 2022-10-14

**Authors:** Yvonne Lott, Anne M. Wöhrmann

**Affiliations:** 1 Hans-Böckler Foundation, Düsseldorf, Germany; 2 Federal Institute for Occupational Safety and Health, Dortmund, Germany

**Keywords:** Dual-earner couples, Path analysis, Spillover and crossover effects, Work–life balance satisfaction, Work–life conflict, Working time demands

## Abstract

To examine the spillover and crossover effects of working time demands (specifically, work contact in leisure time, evening work, and long work hours) on satisfaction with work–life balance among dual-earner couples, path analyses were conducted using data from the 2017/2018 German Family Panel (pairfam; *N* = 1,053 dual-earner couples). Working time demands were measured based on (a) answering work emails/phone calls in leisure time, (b) evening work, and (c) weekly work hours. High working time demands impaired workers’ work–life balance satisfaction due to higher levels of work–life conflict. They indirectly affected partners’ work–life balance satisfaction through two pathways: (a) workers’ and partners’ work–life conflict and (b) workers’ work–life conflict and work–life balance satisfaction. These findings indicate that high working time demands negatively impact the work–life balance satisfaction of workers and their partners because of work–life conflict experienced either by the workers only or by both partners. In an increasingly digitalized labor market, measures are needed to reduce working time demands—and thus work–life conflict—for workers and their partners.

In various labor market sectors, workers face high working time demands. This is due in part to the intensification of work, which results in “work overload” (Kelly & Moen, 2020) and contributes to an increase in working time demands, such as work contact in leisure time, evening work, and long work hours, for employees in jobs with and without work-related use of mobile information and communication technologies (ICTs). Following Bakker and Demerouti (2007), working time demands are those temporal “aspects of the job that require sustained physical and/or psychological (cognitive and emotional) effort or skills and are therefore associated with certain physiological and/or psychological costs” (p. 312). ICTs, such as smartphones, tablets, and laptops, are increasingly available to workers with smart (i.e., flexible) working agreements that allow remote working, as well as to traditional workers who are paid only for hours worked in the office (Ghislieri et al., 2017). This expanded access to ICTs, which has been reinforced by the dramatic increase in remote working for various groups of workers during the COVID-19 pandemic (Abendroth et al., 2022; Felstead, 2022), contributes to “constant connectivity” (Wajcman & Rose, 2011) with high working time demands (Le Bihan & Martin, 2004; Täht & Mills, 2012).

High working time demands challenge workers’ satisfaction with their work–life balance. Work–life balance is a major issue for the quality of life across European countries and is broadly addressed by the European Union’s Social Agenda and its Lisbon Strategy, as well as by the Work–Life Balance Initiative of the European Commission (Szücs et al., 2011). Work–life balance affects individuals’ well-being and health (Greenhaus et al., 2003; Gröpel & Kuhl, 2009; Haar et al., 2014; Shanafelt et al., 2012), as it is a “career value” for employees who want to experience satisfaction and success in fulfilling their commitments in both the work and non-work domains. Thus, satisfaction with work–life balance—which, extending Valcour’s (2007, p. 1512) definition of work–*family* balance, can be defined as “an overall level of contentment resulting from an assessment of one’s degree of success at meeting work” and other life demands—is critical to individuals’ quality of life.

One reason for poor satisfaction with work–life balance among workers with high working time demands might be that these demands increase work–life conflict (Fein & Skinner, 2015; Skinner & Pocock, 2008). Following Bakker et al. (2009), work–life conflict, or spillover, is “a within-person across-domains transmission of strain from one area of life to another” (p. 207)—in this case from work to private life (including partnerships and friendships). Although work–life conflict might mediate the effects of working time demands on work–life balance satisfaction, recent research has neglected to examine it as a possible mediator of the association between these two variables (Albertsen et al., 2008; Arlinghaus et al., 2019; Bjärntoft et al., 2020; Peters et al., 2009). Gao and Jin (2015) examined the mediating effect of work–family conflict on the relationships between job demands (workload, emotional demands, and performance demands), life satisfaction, and job satisfaction in a selective group of workers (i.e., middle-level managers with Chinese state-owned enterprises). McElwain et al. (2005) also analyzed work–family conflict as a mediator of the association between job demands and life satisfaction, job satisfaction, and family satisfaction among full-time professional employees in Canada. However, neither of these studies considered working time demands and their impact on work–life balance satisfaction, nor did they consider work–life conflict and a broader range of workers, although work intensification and constant connectivity is spreading to more and more areas of work (Ghislieri et al., 2017; Kelly & Moen, 2020) and is thus affecting an increasingly diverse group of workers. Hence, the first research question addressed by the present study is whether high working time demands—specifically, work contact in leisure time, evening work and long work hours—impair workers’ work–life balance satisfaction due to work–life conflict.

High working time demands might impair not only individuals’ own work–life balance satisfaction but also that of their partners. This is referred to as a crossover effect. Following Bakker et al. (2009), crossover occurs “when job stress or psychological strain (stress reactions) experienced by one person affects the level of strain of another person in the same social environment” (p. 207). Previous research has shown that working time demands have crossover effects on work engagement (Tonković Grabovac et al., 2016), stress (Bolger et al., 1989; Galambos & Walters, 1992), depression (Westman & Vinoku, 1998; Yoon & Kang, 2016), emotional exhaustion (Zhang et al., 2021), marital satisfaction (Liang, 2015), affect in the family setting (Chan & Margolin, 1994), contribution to housework (Xu et al., 2019), family undermining (Liang, 2015), and the quality of interactions with friends (Rotondi et al., 2017).

By contrast, the extent to which the working time demands of one partner affect the satisfaction with work–life balance of both partners has received less attention. Crossover effects of individuals’ working time demands on work–life balance satisfaction may not only impair the well-being, health, and other outcomes of their partners (Bakker et al., 2009). When experienced by both partners, a poor work–life balance satisfaction may also impair family well-being and relationship quality (Davis et al., 2008; Liang, 2015; Voydanoff, 2007) as well as marital satisfaction (Lavner & Clark, 2017), thereby decreasing family cohesion (Stevens et al., 2006) and negatively affecting children’s behavior (Goldberg & Carlson, 2014) and well-being (Strazdins et al., 2004). This prompts two further research questions: Do workers’ working time demands affect the work–life balance satisfaction of their partners? And, if so, is this due to their own and/or their partners’ work–life conflict?

To answer our research questions, we use large-scale representative data and analyze the relationship between high working time demands and work–life balance satisfaction through work–life conflict. By distinguishing work–life conflict from satisfaction with work–life balance, and by considering work–life conflict as a mediator of the associations between working time demands and work–life balance satisfaction, the present study contributes to filling a gap in existing research in the area of work–life balance, where analyses have often been limited to work–family conflict (Boswell & Olson-Buchanan, 2007; Derks & Bakker, 2014; Wright et al., 2014). However, work–family conflict and work–life balance dissatisfaction are not identical concepts, as work–family conflict does not necessarily result in low satisfaction with work–life balance for all individuals (Szücs et al., 2011; Valcour, 2007), whose experiences of conflict between the work and family domains may vary (Thilagavathy & Geetha, 2020), or for whom levels of conflict may fluctuate even from day to day (McDowall & Kinman, 2017). Moreover, individuals’ preferences have become more diverse, and workers may place value on other non-work activities beyond family, such as leisure, personal time, voluntary work, or political engagement (Kelliher et al., 2019). According to life course theoretical approaches, individual life-course patterns are influenced by the dynamics of social groups beyond the family (Mayer, 2004), such as workplaces, organizations, neighborhoods and communities (Courtright et al., 2016; Wilson et al., 2018). The present study addresses these issues by extending the analysis of work–*life* balance satisfaction and work–*life* conflict beyond work–family conflict. We thus consider other areas of life that can also be negatively affected by work, such as the “personal domain, which includes activities one pursues because of his or her own interests (e.g., friends, hobbies, community)” (Wilson & Baumann, 2015, p. 235). In doing so, we take account of the fact that the relevance of work and private life differs across individuals, that “private life encompasses more than the family role” (Abendroth & den Dulk, 2011), and that activities in various domains may be mutually enriching (McDowall & Kinman, 2017).

The most important contributions of the present study to existing research are as follows: First, whereas studies on working time demands generally focus on one dimension of working time, for example, flexibility (Derks & Bakker, 2014; Wright et al., 2014), this study considers all three dimensions (Vieten et al., 2021)—namely, the flexibility of working time (operationalized as work contact in leisure time), the timing of work (operationalized as evening work), and the duration of working time (operationalized as long work hours). In doing so, working time demands are considered in their different variations. Second, spillover and crossover effects of various working time demands on satisfaction with work–life balance are analyzed, thereby also addressing the importance of individuals’ work arrangements for their partners. As the present study takes into account working time demands that are not necessarily related to the use of ICTs, and it thus also includes workers who do not use such technologies, we complement previous research (e.g., Carlson et al., 2018) showing that the work-related use of ICTs during family time negatively affects partners’ work lives. By considering the three dimensions of working time, and not just the use of ICTs, this study focuses on a much broader workforce that is exposed to high working time demands with and without the work-related use of ICTs, and takes an in-depth analytical perspective, asking why high working time demands in their three dimensions might impair individuals’ and their partners’ work–life balance satisfaction.

## Intraindividual effects of working time demands at the work–life interface

Following Valcour (2007), satisfaction with work–family balance includes an affective and a cognitive component:

The cognitive component involves an appraisal of one’s degree of success in meeting the multiple demands of work and family roles. The affective component entails a positive feeling or emotional state resulting from that appraisal. Satisfaction with work–family balance results from individuals’ assessment that they have adequate resources to effectively respond to the demands of their work and family roles. (Valcour, 2007, p. 1513).

This can be applied mutatis mutandis to satisfaction with work–*life* balance.

According to Voydanoff’s (2007) conceptual model of work, family, and community, a domain is characterized by basic organization and by boundaries—that is, by structure, which “in paid work encompasses organizational characteristics, extrinsic characteristics, timing, and spatial location” (p. 5). Applying this definition to other life domains, *timing* refers to the amount of time individuals spend on activities in a particular domain and to when these activities are performed, and *spatial location* refers to where the activities are performed (Voydanoff, 2007, p. 5). Working time demands may increase the permeability of the temporal, physical, and psychological boundaries between the work and private life domains—that is, “the degree to which elements from other domains may enter” (Clark, 2000, p. 756)—and may complicate the management and maintenance of boundaries between work and private life. This may result in distractions from work and private life, respectively, thereby hindering the fulfilment of both work and non-work roles (Jostell & Hemlin, 2018) and the psychological detachment from work during leisure time (Sonnentag, 2012).

Besides blurring the boundaries between the work and non-work domains, working time demands may lead to role pressure incompatibility (Greenhaus & Beutell, 1985), which arises when there is a conflict between the norms and requirements of the respective roles. Working time demands “may make it physically impossible to comply with expectations of another role” (Greenhaus & Beutell, 1985, p. 78) because they may interfere with the “social rhythm” of activities in the evenings and on weekends, “which are still considered the most valuable times for social and family interactions” (Arlinghaus et al., 2019, p. 186). The pressures associated with working time demands may also “produce preoccupation with the work role even when one is physically attempting to meet the demands of another role” (Greenhaus & Beutell, 1985, p. 78), and may thus diminish the resources necessary to respond to the demands of roles in non-work domains.

Both the blurring of the boundaries between the work and non-work domains and role pressure incompatibility may lead to work–life conflict. Extending the definition of work–family conflict (Greenhaus & Beutell, 1985; Greenhaus & Parasuraman, 1987), work–life conflict refers to conflict between individuals’ roles at work and in their private lives; it may be time-based, strain-based, or behavior-based (Greenhaus & Beutell, 1985). Behavior-based conflict exists when behavior that is appropriate for the work role is inappropriate for roles in other domains; strain-based conflict involves emotional interference between the work role and other life roles; time-based conflict occurs when work-related time demands hinder the fulfillment of requirements of other life roles. In the present study, we focus on time-based and strain-based work–life conflict.

Applying these theoretical considerations to the three dimensions of working time (i.e., duration, timing, and flexibility; Vieten et al., 2021), this means that for workers with working time flexibility who engage in work contact in leisure time, the physical and temporal boundaries between the work and non-work domains become blurred, and the distinctions between the work role and other life roles become unclear (Schieman & Young, 2013). These workers may also experience role pressure incompatibility because they are preoccupied with the work role during family or other social interactions. Previous research has shown that individuals who have work contact in leisure time are more likely to be distressed, feel guilty, have sleep problems, and feel less recovered (Kim et al., 2019; Schieman & Glavin, 2008; Schieman & Young, 2013; Vieten et al., 2021)—factors that impair physical and mental health (Burchell et al., 2002; Robinson & Godbey, 1997; Roxburgh, 2004; Shields, 1999) and negatively affect family life (Green, 2004; Kattenbach et al., 2010; Macky & Boxall, 2008; Roxburgh, 2004). Recent studies have also shown that work-related smartphone use in the evenings hinders engagement in recovery activities (Derks et al., 2014), is related to emotional exhaustion (Xie et al., 2018), and impairs well-being (Gombert et al., 2018). Work contact in leisure time also increases work–family conflict and work–life conflict (Boswell & Olson-Buchanan, 2007; Derks & Bakker, 2014; Eby et al., 2005; Ghislieri et al., 2017; Wright et al., 2014), and it is related to less satisfaction with work–life balance (Brauner et al., 2021).

Nonstandard work schedules, which represent the second dimension of working time (i.e., timing), are highly incompatible with the social rhythm of family and other social activities, which tend to take place mainly in the evenings and on weekends. They also diminish resources needed to respond to the demands of roles in the non-work domains. Due to this role pressure incompatibility, nonstandard work schedules may impair workers’ involvement in family life and their responsiveness to their children (Bünning & Pollmann-Schult, 2016), have negative effects on marital stability and children’s behavior and well-being (Strazdins et al., 2004), and are related to work–family conflict (Eby et al., 2005; Wöhrmann et al., 2020).

Like workers who work in the evenings and at weekends, those who work longer hours may also experience role pressure incompatibility because their nonstandard work schedules likely detract from family time, and they have less time for recovery from work (Schiller et al., 2018). Thus, long work hours, which represent the third dimension of working time (i.e., duration), may lead to exhaustion, distress, and mental and physical health problems (Bakker & Geurts, 2004; Kattenbach et al., 2010; Krause et al., 2005; Yang et al., 2021), which may in turn reduce the quality of life at home (Macky & Boxall, 2008; Roxburgh, 2004). Employees who work longer hours have fewer resources and less time to perform activities in the family domain (Crouter, 1984; Kopelman et al., 1983). As a consequence, conflicts arise between the work and family domains (Eby et al., 2005; Skinner & Pocock, 2008; Steiber, 2009; White et al., 2003), and between work and non-work domains in general (Fein & Skinner, 2015; Skinner & Pocock, 2008).

Because workers are likely to experience higher levels of work–life conflict when they have work contact in leisure time, nonstandard work schedules (e.g., evening work), and long work hours, individuals with these working time demands might be less satisfied with their work–life balance. Following Voydanoff (2007), people evaluate their success in coping with work and life demands. If they feel they are unsuccessful—for example, if they experience conflict between their work and their private lives—this leads to a negative emotional state, namely, lower satisfaction. Previous studies have shown, for example, that work–family conflict negatively affects workers’ domain satisfaction and overall life satisfaction (Allen et al., 2000). By extension, work–life conflict may also have a negative impact on workers’ satisfaction with their work–life balance, a concept that goes beyond the family domain to include also the personal domain (Wilson & Baumann, 2015).

As working time demands are associated with work–life conflict, and work–life conflict is associated with employees’ domain-specific and general life satisfaction and may also be associated with work–life balance satisfaction, work–life conflict might mediate the effect of high working time demands on work–life balance satisfaction. Some studies have shown that the associations between job demands (i.e., workload, emotional demands, performance demands) and life and job satisfaction are mediated by work–family conflict (Gao & Jin, 2015; McElwain et al., 2005). However, work–life conflict might be an even more critical mediator than work–family conflict for the association between working time demands and work–life balance satisfaction, because work–life conflict also encompasses the personal domain (Wilson & Baumann, 2015) that is, the area of private life in which individuals pursue their own interests, “(e.g., friends, hobbies, community)” (p. 235). For example, individuals who work long hours may not have enough time to socialize with friends (time-based work–life conflict), or they may enjoy the company of friends less due to work-related stress (strain-based work–life conflict), and, as a result, they may be less satisfied with their work–life balance. Following from this, our first hypothesis is:

- *Hypothesis 1*: High working time demands—specifically, work contact in leisure time, evening work, and long work hours—indirectly impair workers’ work–life balance satisfaction through work–life conflict.

## Crossover effects of working time demands at the work–life interface

Individuals in close relationships—for example, with family members or partners—“are interdependent in the outcomes of interaction” (Thibaut & Kelley, 2017, p. *v*). They “emit behavior in each other’s presence, they create products for each other, or they communicate with each other” (Thibaut & Kelley, 2017, p. 10), and “they can influence one another in their thoughts, emotions and behaviors” (Schnettler et al., 2020b, p. 2). Because “closely related partners who care for each other and share most of their lives” (Westman & Etzion, 2005) pay close attention to one another and perceive themselves as interrelated to each other, they feel with and feel into the other (Bakker et al., 2009, p. 211). Following the spillover–crossover model (Bakker et al., 2009), this empathy leads to susceptibility to emotional contagion of negative and positive emotion—that is, to the crossover of negative and positive emotions to the other. Emotional contagion in couples has been found for psychological health problems (Katz et al., 2011; Thomeer et al., 2013; Wang et al., 2017b), work engagement (Bakker et al., 2005), and observational learning of goal regulation processes (Kappes & Thomsen, 2020). Work–family conflict has been found to cross over to partners, who, due to empathy and emotional contagion, also perceive conflicts between the work and family domains (Hammer et al., 1997; Westman & Etzion, 2005). Thus, work–life conflict can also cross over to partners.

Another explanation for the crossover of work–life conflict is that it represents additional stress. Regarding work–family conflict, it has been found that individuals’ work–family conflict is related to energy deficits and time deficits (ten Brummelhuis et al., 2010) and may therefore “create an additional source of stress” for their partners (Hammer et al., 1997, p. 189), who may likewise experience psychological distress and anger (Young et al., 2014). For example, individuals whose partners experience work–life conflict due to work contact in leisure time, evening work, or long work hours may be reminded of their own work and, as a result, experience the blurring of boundaries between work and family. They may feel pressured to work more, too, or they may feel guilty for not doing so, which may increase their own work-related stress. They may also increase their own workload due to the working time demands of others, in the sense of behavioral contagion. Christakis and Fowler (2013) found behavioral contagion in couples for health-related behavior such as smoking. As a consequence, individuals whose partners experience work–life conflict may be less able to manage work-related stress and to meet the demands of their non-work roles, with the result that they experience higher levels of work–life conflict themselves (Westman & Etzion, 2005). These assumptions are supported by results of previous research showing that, in dual-earner couples, both partners are very likely to experience work–family conflict simultaneously (Matias et al., 2017; Vieira et al., 2016). The spillover–crossover model and the additional-stress perspective suggest that individuals’ working time demands might indirectly impair their partners’ work–life balance because working time demands increase individuals’ work–life conflict, which in turn increases their partners’ work–life conflict.

Furthermore, Bakker et al. (2009) highlighted the fact that not only negative but also positive emotions can cross over to intimate partners. Indeed, Rodríguez-Muñoz et al. (2014) and Christakis and Fowler (2013) showed that workers’ happiness was emotionally contagious for their partners. Following from this, individuals’ satisfaction with their work–life balance may contribute to their partners’ work–life balance satisfaction due to empathy and emotional contagion. Previous studies have also found crossover effects of domain satisfaction—for example, job satisfaction and satisfaction with family life—and overall life satisfaction in couples (Demerouti et al., 2001; Dobewall et al., 2019; Schnettler et al., 2020a, 2020b). Therefore, workers’ satisfaction with their work–life balance might also affect their partners’ work–life balance satisfaction. Conversely, workers’ dissatisfaction with their work–life balance might impair their partners’ work–life balance satisfaction. Consequently, workers’ working time demands might indirectly impair their partners’ work–life balance satisfaction, because working time demands increase workers’ work–life conflict, which in turn decreases their work–life balance satisfaction.

Thus, working time demands might indirectly affect partners’ work–life balance satisfaction via two pathways: (a) workers’ and partners’ work–life conflict and (b) workers’ work–life conflict and work–life balance satisfaction. To illustrate this with an example: Because work contact during leisure time blurs the boundary between work and private life and contributes to role pressure incompatibility, it increases the conflict between work and life. This affects partners’ satisfaction with work–life balance in two ways: (a) work–life conflict in workers leads to work–life conflict in partners, thereby affecting partners’ satisfaction with their work–life balance; and (b) work–life conflict in workers affects workers’ satisfaction with their work–life balance, which in turn affects partners’ satisfaction with their work–life balance. The same applies to evening work and long work hours, which contribute to role pressure incompatibility and indirectly affect partners’ satisfaction with work–life balance through (a) workers’ work–life conflict and their own work–life conflict and (b) workers’ work–life conflict and work–life balance satisfaction. Thus, we hypothesize:

- *Hypothesis 2a:*
- Workers’ high working time demands—specifically, work contact in leisure time, evening work, and long work hours—indirectly impair their partners’ work–life balance satisfaction through workers’ work–life conflict and partners’ work–life conflict.
- *Hypothesis 2b:*
- Workers’ high working time demands—specifically, work contact in leisure time, evening work, and long work hours—indirectly impair their partners’ work–life balance satisfaction through workers’ work–life conflict and work–life balance (dis)satisfaction.

**Fig. 1:**
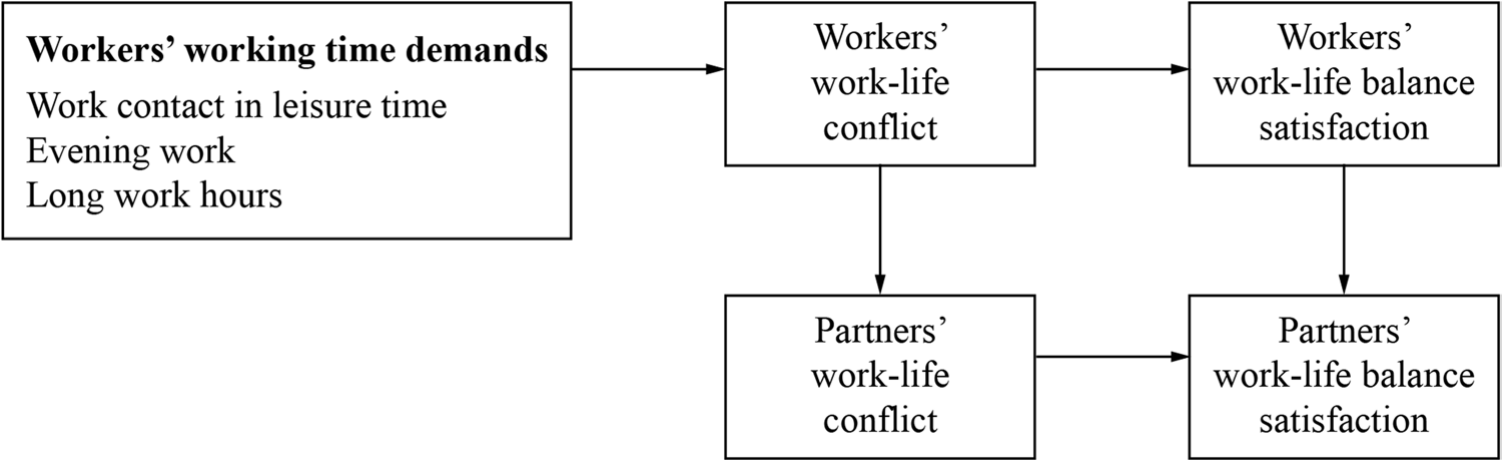
Path model of hypothesized working time demands, work–life conflict, and work–life balance satisfaction in dual-earner couples

Figure 1 shows the hypothesized relationships between workers’ working time demands and workers’ and their partners’ work–life conflict and work–life balance satisfaction.

## Method

### Data and sample

We used data from the 10th wave of the German Family Panel (pairfam; 2017/2018; Brüderl et al., 2019), a study that researches intimate relationships and family dynamics in Germany. The panel study is based on a random sample of the population in Germany drawn from municipal population registers, without restrictions on the occupations of participants; the sample for Wave 10 comprised participants from three birth cohorts: 1991–1993, 1981–1983, and 1971–1973 (Brüderl et al., 2019; Huinink et al., 2011). The panel was extended to include an additional sample of eastern German respondents, who receive the same questionnaires as the respondents in the initial sample. Pairfam has a multi-actor design, whereby the “anchor persons” are asked to consent to their partners being interviewed. The anchors take part in computer-assisted personal interviews that last about one hour. Half of the participants with a partner gave their consent to their partners being interviewed. These partners receive a modified, shorter paper-and-pencil questionnaire. The anchor population comprises persons living in private households in the Federal Republic of Germany. The data set of the survey wave used in the present study (2017/2018) contains data from 4,750 anchors and 1,799 partners. We used a subsample that included all 1,053 dual-earner couples who provided data on the study variables. About half of the sample (51%) were female anchors with male partners. The mean age of the anchors (range 24–47 years) and their partners (range 22–60 years) was 41 years. Both groups had 14 years of education, on average. About two-thirds (68%) of the anchors were full-time employed; in 39% of the couples, both partners were employed full-time. Most participants were married (87%) and had children (82%). Using a random sample, no restrictions regarding occupations of participants were applied.

### Measures

#### Working time demands: work contact in leisure time, evening work, and long work hours

Following the job demands scale proposed by Rosin and Korabik (1991), we measured three work-related behaviors: work contact in leisure time, evening work, and long work hours. Working time demands in terms of work contact in leisure time (flexibility), evening work (timing), and long work hours (duration of working time) were measured with one question or item each. Work contact in leisure time was measured with the item: “I answer work messages during my leisure time – e.g., emails or phone calls.” to be answered on a 5-point Likert scale ranging from 1 (*disagree completely*) to 5 (*agree completely*). Evening work was measured with the question: “Do you frequently work after 7 p.m.? (yes/no).” Long work hours was measured with the question: “What, on average, are your actual weekly working hours, including overtime? (hours)?” These items or questions were administered only to the anchors.

#### Work–life conflict

In line with Fein and Skinner (2015) and Skinner and Pocock (2008), time-based and strain-based work–life conflict was measured with the following items that focused on activities and time spent with friends, partner, and/or family or on activities in private life in general: “Due to the workload at work, in training or in my studies, I don’t have enough time for my private life.”; “Even if I do something with friends, partner or family, I often have to think about work.”; “After the stresses of work, I find it difficult to relax at home and/or to relax and/or enjoy my free time with others.”; “My work keeps me more from doing things with friends, partner and family than is comfortable for me.” The response categories for each item ranged from 1 (*not at all*) to 5 (*absolutely*). Cronbach’s alpha was 0.67 for the anchors and 0.98 for the partners. The anchors and their partners responded to these items separately. Mean scale scores were calculated for anchors’ and partners’ work–life conflict.

#### Satisfaction with work–life balance

Information on satisfaction with work–life balance was available for both partners, who answered the following survey question separately: “How satisfied are you with the proportion of time that you spend on your job relative to the time that you spend on your personal life?” Satisfaction with work–life balance was used as a continuous dependent variable and measured on a scale from 0 (*very dissatisfied*) to 10 (*very satisfied*). Current research shows that constructs in the organizational sciences can often be assessed reliably and validly with a single item (Matthews et al., 2022). Further, it is common to measure satisfaction with work–life balance with a single item (Saltzstein et al., 2001; Wöhrmann et al., 2021).

Inspection of the correlation matrix (Bagozzi et al., 1991), as well as Harman’s single-factor test (Fuller et al., 2016) gave no indication of common-method bias with regard to the items used in this study. The maximum correlation between study variables was 0.60, and a principal component analysis revealed that one factor explained 29% of total variance, which is below the 50% threshold.

#### Control variables

Because workers in full-time jobs have a higher risk of work–family conflict (Michel et al., 2011) due to higher workload (Moen & Yu, 2000), we controlled for whether the anchor experienced high workload in the job (“I often have to deal with too heavy workloads.”; 5-point Likert scale ranging from 1 = *completely disagree* to 5 = *completely agree*) and whether their partner worked full-time. To account for family responsibilities, we controlled for whether at least one child was living in the household. To account for possible gender role effects with regard to the work–life interface, we also controlled for the anchor’s sex. Earlier research has shown that parental status and gender are important aspects in the context of the work–life interface (Abendroth et al., 2022; Lott & Chung, 2016; Lott, 2020b). As working part-time rather than full-time can make a meaningful difference with regard to the work–life interface (Borgmann et al., 2019), we conducted an additional exploratory analysis in which we stratified our model for full- and part-time employed anchors. This also allowed us to interpret the role of the number of work hours within these two groups as hypothesized within our study model.

### Data analysis

We conducted path analyses using Mplus Version 7.4 (Muthén & Muthén, 2015). We modeled missing values using a maximum likelihood estimator (Wang et al., 2017a). When testing indirect effects (Preacher & Hayes, 2008), we used bootstrapping (with 10,000 draws) to account for any deviations from normality. Model fit was evaluated with the root-mean-square error of approximation (RMSEA; Steiger, 1990) as an absolute fit index, and the comparative fit index (CFI; Bentler, 1990) as an incremental fit index. Although we report the chi-square value, we did not use it for the interpretation of model fit, because it is sensitive to sample size. In line with the wording of our hypotheses, anchor persons are referred to as “workers” when presenting and discussing the results.

## Results

### Preliminary analysis

Means, standard deviations, and correlations of the study variables are presented in Table 1. Working time demands represented by answering work emails/phone calls in leisure time, evening work, and duration of weekly work hours were positively related to workers’ work–life conflict. Workers’ and partners’ work–life conflict and satisfaction with work–life balance were all interrelated. The inspection of the baseline model (fully identified) to evaluate the explanatory impact of the control variables showed that they explained more variance in workers’ work–life conflict (*R*^2^ = 0.23) and in workers’ work–life balance satisfaction (*R*^2^ = 0.12) than in partners’ work–life conflict (*R*^2^ = 0.07) and in partners’ work–life balance statisfaction (*R*^2^ = 0.05).

**Table 1:** Correlations, means, and standard deviations of the study variables

Variable	*M* (*SD*)	1	2	3	4	5	6	7	8	9	10	11
1. Workers’ sex (female)	.51 (.50)											
2. At least one child in household	.82 (.38)	.02										
3. Workers’ workload	3.15 (1.15)	** − **.05	** − **.03									
4. Full-time job (workers)	.68 (.47)	** − **.57***	-.17***	-.11***								
5. Full-time job (partners)	.68 (.47)	.54***	** − **.16***	-.04	-.33***							
6. Answering work emails/phone calls in leisure time (workers)	2.51 (1.48)	** − **.04	-.02***	.09**	.09**	** − **.05						
7. Evening work (workers)	.35 (.48)	** − **.08*	** − **.01	.11***	.09**	** − **.04	.23***					
8. Weekly work hours (workers)	36.32 (12.31)	–.52***	** − **.14***	.28***	.72***	-.31***	.17***	.18***				
9. Workers’ work–life conflict	2.31 (.89)	** − **.11**	-.09**	.45***	.20***	** − **.10**	.30***	.25***	.40***			
10. Partners’ work–life conflict	2.49 (.95)	.15***	-.10**	.05	–.09***	.24***	.01	.01	** − **.08*	.11**		
11. Workers’ work–life balance satisfaction	6.32 (2.10)	.08**	.00	-.35***	–.16***	.06	** − **.08*	–.19***	–.32***	–.60***	–.14***	
12. Partners’ work–life balance satisfaction	5.93 (2.43)	–.15***	.07*	–.04	.06*	–.21***	.03	–.00	.06*	** − **.07*	–.57***	.14***

*Note. N* = 1,053 dual-earner couples

^*^p < .05. **p < .01. ***p < .001

Structural coefficients suggested that workers’ high workload, partners’ full-time employment, and at least one child living in the household were unfavorably related to workers’ and partners’ work–life conflict. Further, workers’ workload was negatively related to workers’ work–life balance satisfaction, and partners’ full-time employment was negatively related to partners’ work–life balance satisfaction. The respondent’s sex was unrelated to work–life conflict and satisfaction with work–life balance. The pattern of the results of hypotheses testing presented below did not change with or without the inclusion of control variables in the model.

### Hypotheses testing

**Fig. 2:**
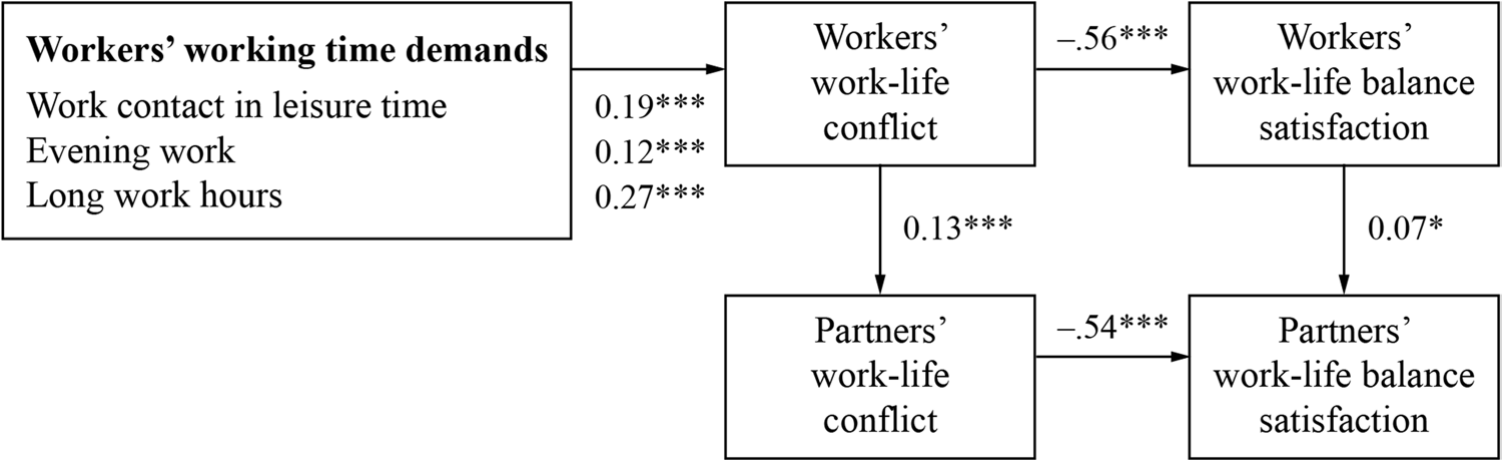
Direct effects between working time demands, work–life conflict, and work–life balance satisfaction. Note: *N* = 1,053 dual-earner couples; standardized coefficients are reported. * *p* < .05. ***p < .001

The model showed a good fit to the data, χ^2^(11) = 56.60; RMSEA = 0.06; CFI = 0.97; standardized root-mean-square residual (SRMR) = 0.02. The structural relationships (direct effects) between the study variables are shown in Fig. 2; the findings regarding the hypothesized indirect effects between working time demands and workers’ and partners’ work–life balance satisfaction are presented in Table 2. All hypothesized indirect effects were significant. More specifically, we hypothesized that workers’ working time demands were indirectly related to their satisfaction with work–life balance through their work–life conflict (Hypothesis 1). This hypothesis could be confirmed: Workers’ higher working time demands in terms of answering work emails/phone calls in leisure time, evening work, and number of weekly work hours were indirectly related to less satisfaction with their work–life balance because working time demands were positively related to workers’ work–life conflict, which in turn was negatively related to their satisfaction with their work–life balance.

**Table 2:** Indirect effects of workers’ working time demands on workers’ and partners’ work–life balance satisfaction

Workers’ working time demands	Workers’ WLBS through workers’ WLC (H1)	Partners’ WLBS through workers’ WLC and partners’ WLC (H2a)	Partners’ WLBS through workers’ WLC and workers’ WLBS (H2b)
Answering work emails/phone calls in leisure time	− .11*** (.02) [− .14; − .08]	− .01** (.00) [− .02; − .00]	− .01* (.00) [− .02; − .00]
Evening work	− .07*** (.02) [− .11; − .04]	− .01** (.00) [− .02; − .00]	− .01* (.00) [− .01; − .00]
Weekly work hours	− .16*** (.02) [− .20; − .12]	− .02*** (.01) [− .03; − .01]	− .01* (.01) [− .02; -.00]

*Note. N* = 1,053 dual-earner couples. WLC = work–life conflict; WLBS = work–life balance satisfaction; H = hypothesis. The table presents standardized coefficients, standard errors in parentheses, and lower and upper levels of 95% confidence intervals (bootstrapped) in brackets.

^*^p < .05. **p < .01. ***p < .001

Based on the considerations in Hypothesis 1, we further hypothesized that workers’ working time demands were indirectly related to partners’ satisfaction with their work–life balance through workers’ work–life conflict and partners’ work–life conflict (Hypothesis 2a) and through workers’ work–life conflict and workers’ (dis)satisfaction with work–life balance (Hypothesis 2b). Both hypotheses were confirmed. Workers’ work–life conflict was positively related to partners’ work–life conflict, which in turn was negatively related to partners’ satisfaction with their work–life balance. Moreover, workers’ satisfaction with their work–life balance was positively related to partners’ satisfaction with their work–life balance. Thus, workers’ working time demands were indirectly and negatively related to partners’ satisfaction with their work–life balance through these two paths.

The study variables explained variance in the outcome variables over and above the control variables: workers’ satisfaction with their work–life balance: *R*^2^ = 0.26; partners’ satisfaction with their work–life balance: *R*^2^ = 0.28. Working time demands explained 13% of the variance in workers’ work–life conflict.

**Fig. 3:**
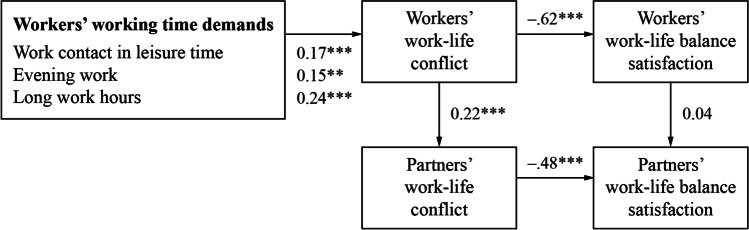
Direct effects between workers’ working time demands, work–life conflict, and work–life balance satisfaction for part-time workers. Note: *N* = 338 dual-earner couples; standardized coefficients are reported. * *p* < .05. ***p < .001

**Fig. 4:**
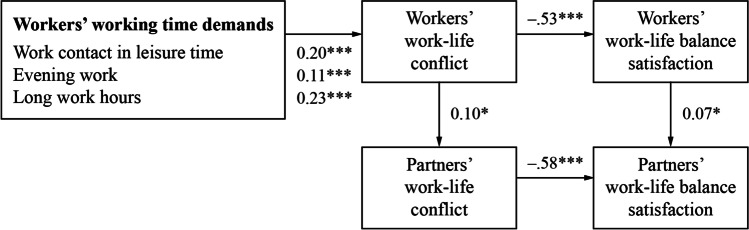
Direct effects between workers’ working time demands, work–life conflict, and work–life balance satisfaction for full-time workers. Note: *N* = 719 dual-earner couples; standardized coefficients are reported. * *p* < .05. ***p < .001

Exploratory path analyses for a stratified sample of full-time workers (*N* = 719 dual-earner couples) and part-time workers (*N* = 338 dual-earner couples) showed a good model fit to the data: χ^2^(22) = 66.75; RMSEA = 0.06; CFI = 0.97; SRMR = 0.02 (see Tables 3 and 4 and Figs. 3 and 4 in the Appendix). The results patterns of direct effects between the study variables did not differ significantly between groups, except that for part-time workers the relationship between their own and their partners’ work–life balance satisfaction was not significant. Thus, Hypothesis 2b, which assumed an indirect effect of workers’ working time demands on partners’ work–life balance satisfaction via workers’ work–life conflict and workers’ work–life balance (dis)satisfaction could not be supported for part-time workers. However, Hypotheses 1 and 2a were fully supported for that group.

Hypothesis 1 was also supported for full-time workers, as working time demands were significantly and indirectly related to their work–life balance satisfaction via work–life conflict. The direct relationships between full-time workers’ work–life balance satisfaction and partners’ work–life balance satisfaction, as well as between full-time workers’ work–life conflict and partners’ work–life conflict were both statistically significant but weak. For the indirect effects of full-time workers’ working time demands on partners’ work–life balance satisfaction, we found significant effects through workers’ work–life conflict and partners’ work–life conflict for work contact in leisure time and for evening work but not for long work hours. Thus, Hypotheses 1 and 2a were supported for both part-time and full-time workers, although evidence for full-time workers is weak and was found only for leisure time and evening work.

## Discussion

The aim of the present study was to examine the spillover and crossover effects of working time demands—specifically, work contact in leisure time, evening work, and long work hours—on work–life balance satisfaction in dual-earner couples. We asked (a) whether high working time demands impaired work–life balance satisfaction due to work–life conflict; and (b) whether workers’ high working time demands also affected their partners’ satisfaction with work–life balance through workers’ and/or their partners’ work–life conflict.

The results support previous findings showing that working time demands lead to work–life conflict (Boswell & Olson-Buchanan, 2007; Derks & Bakker, 2014; Wright et al., 2014). They further show that this impairs workers’ work–life balance satisfaction. Moreover, the analyses revealed that the various dimensions of working time—namely, its flexibility, timing, and duration (Vieten et al., 2021)—affect satisfaction with work–life balance. Because high working time demands in these three dimensions are associated with the blurring of the boundary between the work and non-work domains (work contact in leisure time) and with role pressure incompatibility (long work hours, evening work, and work contact in leisure time), work can more easily spill over into non-work domains and/or diminish the resources that are necessary to fulfill private-life roles (Greenhaus & Beutell, 1985). As a result, employees experience work–life conflict. This supports Voydanoff’s (2007) conceptual model of work, family, and community, according to which domains are characterized inter alia by timing and spatial location (Voydanoff, 2007, p. 5). Work in the evenings and long work hours reduce the resources that are necessary to fulfill private-life roles, because individuals invest a relatively large amount of time in the work domain and are active in that domain during family and other social times (timing). Moreover, with work contact in leisure time, work spills over into non-work domains, and individuals are active in the work domain even when they are in the family domain or in other domains of their private lives (spatial location). In addition, the analyses also revealed that the spillover and crossover effects were stronger among part-time workers and their partners. For full-time workers, the spillover and crossover effects were weaker. For this group, long work hours did not have crossover effects on partners’ work–life balance satisfaction, possibly due to lower variance of work hours among full-time workers compared with part-time workers.

Finally, the present study shows that the three dimensions of working time demands lead to crossover effects on satisfaction with work–life balance in couples, and that partners are at risk of having a poorer work–life balance satisfaction due to workers’ high working time demands for two reasons—namely, (a) because partners also experience work–life conflict or (b) because partners’ work–life balance satisfaction is affected by their significant others’ work–life balance satisfaction. The present analyses add to recent studies on crossover effects of working time demands in couples (Bolger et al., 1989; Chan & Margolin, 1994; Liang, 2015; Rotondi et al., 2017; Westman & Vinoku, 1998; Yoon & Kang, 2016) by explaining why workers’ working time demands impair their own as well as their partners’ work–life balance satisfaction. Workers’ work–life conflict is a major reason for poor work–life balance satisfaction on the part of both partners. This supports the spillover–crossover model (Bakker et al., 2009) and confirms the assumption that not only negative but also positive emotions such as work–life balance satisfaction cross over to closely related persons (Bakker et al., 2009).

### Limitations and future research

The present study has a number of limitations. First—and foremost—information on partners’ workplace characteristics was incomplete, and partners’ working time demands were not measured. These factors might further explain the crossover effects in couples. High working time demands, for example, might be even more problematic if experienced by both partners. Some intra-couple constellations of working time demands might even reduce work–life conflict—for example, when one partner’s very low working time demands balance out the high working time demands of the other partner. In addition, partners’ job resources, such as job autonomy, might not only buffer the crossover effects but also the intra-individual effects of working time demands on work–life balance satisfaction.

Second, due to the relatively small number of couples for whom information on both partners was available, the present study applied cross-sectional data analyses and therefore did not take individual-self selection and time-constant unobserved heterogeneity into account. Future research should use more extensive dyadic panel data that include broad information on the work characteristics, job resources, and job demands of both partners.

Third, although the causes of high work demands may vary depending on the industry and the job type, this could not be considered in the present study due to the limited data on workers’ and their partners’ workplace characteristics. More extensive data are needed to examine possible variations in the workforce.

Fourth, whereas some studies have focused on working time demands that are related to telework and after-hours communication/availability requirements and expectations (Day et al., 2012; Dettmers et al., 2016; Park et al., 2020; Piszczek, 2017), the present study considered, in line with previous research (Carlson et al., 2018; McElwain et al., 2005; Rosin & Korabik, 1991), work-related behavior—namely, work contact in leisure time, evening work, and long work hours. In order to link this work-related behavior to workplace requirements and expectations, dyadic panel data that also include workplace characteristics must therefore be used in future research.

And finally, fifth, due to the cross-sectional nature of the data, all conclusions are only correlational, not causal. Therefore, reverse relationships cannot be ruled out, especially because in this study working time demands were subjective assessments. Thus, interpretations of items used in the study, such as assessment on a frequency scale, may have differed among respondents. To determine the effects of working time demands on workers’ and their partners’ work–life conflict and work–life balance satisfaction, future studies could apply an intervention design in which a change in objective working time demands is applied.

### Implications for theory and research

The finding that work–life conflict mediates the relationship between working time demands and satisfaction with work–life balance suggests that working time demands affect not only workers’ family domain but also their personal domain (e.g., friends, hobbies, community; Wilson & Baumann, 2015), and that work–life conflict is an important mediator of the effect of workers’ working time demands on their work–life balance satisfaction. Workers with high working time demands do not have enough time for their personal lives, for example, to meet with friends (time-based work–life conflict), or they enjoy the company of friends less due to work-related stress (strain-based work–life conflict). As a result, they are less satisfied with their work–life balance. This finding supports Kelliher et al. (2019), who argued that workers place value on private activities beyond family, such as hobbies or volunteering. It also supports life course theoretical approaches (e.g., Mayer, 2004) that see individuals’ life courses as being embedded in various groups beyond the family, such as circles of friends, neighborhoods, and communities (Courtright et al., 2016; Wilson et al., 2018).

Moreover, the finding that spillover and crossover effects of working time demands on work–life balance satisfaction exist, supports Bakker et al.’s (2009) spillover–crossover model and suggests that working time demands that are not part of an individual’s own job but of that of their partner, nevertheless have an impact on that individual’s work–life conflict and thus on their satisfaction with their work–life balance. Following the spillover–crossover model, individuals in close relationships who feel with and feel into the other (Bakker et al., 2009) allow themselves be “infected” by the other’s work–life conflict. As a result, they themselves experience work–life conflict and, as a consequence, are less satisfied with their work–life balance. However, the present study also proposed a complementary explanation for these crossover effects, namely, the additional-stress perspective whereby an individual’s conflict between the work and private-life domains “creates an additional source of stress” (Hammer et al., 1997, p. 189) for their partner, who in turn is less able to cope with their own work-related stress and to meet the demands of their private-life roles. For example, individuals whose partners experience work–life conflict may themselves have greater difficulties drawing and managing the boundary between work and private life, and may experience more role pressure incompatibility through greater work-related stress because the other’s preoccupation with work during leisure time reminds them of their own work, contributes to a feeling that they should be working more, too, and/or makes them feel guilty for not doing so. Moreover, behavioral contagion whereby an individual’s work-behavior is imitated by their partner may occur.

Future research is therefore needed to further reveal the mechanisms of crossover in close relationships in order to understand why employees allow themselves be “infected” by their partners’ negative work outcomes. Because the risk of emotional or behavior contagion may vary for individuals depending on their personality traits, relationship quality, and self-esteem, future research should also take these characteristics into account. By doing so, vulnerable groups of workers and partners can be detected and adequate measures implemented to overcome these problems. This is of relevance especially with regard to the increasing use of ICTs and the prevalence of working from home during and probably after the COVID-19 pandemic, which may pose a threat to employees’ health and well-being (Felstead, 2022). Gaining more empirical insights into the crossover process in close relationships may also help to develop the spillover–crossover model further and to integrate the additional-stress perspective and the concept of behavioral contagion.

### Implications for practice

The present study underscores the need for individual as well as company-level measures to prevent high working time demands—especially work contact in leisure time, evening work, and long work hours—and thus work–life conflict. Occupational health and safety policies must make workers aware of the risks of their unhealthy work behavior for their partners and other family members. The message should be that although workers may be okay with high working time demands for career reasons or because they work for an organization that has an ideal worker culture (Williams et al., 2013), their partners may not. Rather, their partners are likely to experience a poor work–life balance, which can lead to physical and mental health problems. The more boundary-spanning demands exist, and the more the boundaries between the work and family domains become permeable, the greater the need for individual measures and occupational health and safety policies that take into account the work–life balance outcomes not only of workers but also of their partners and other family members. This is of special importance during the COVID-19 pandemic, where a great number of workers are working remotely, thereby putting not only their own but also their partners’ work–life balance at risk (Felstead, 2022). To manage the boundary between work and private life, and to limit time investment in work, workers need specific individual strategies to cope with these challenges. For example, they could participate in coaching or training that has been specifically designed for this context and includes different aspects related to boundary management (e.g., Rexroth et al., 2017).

Relevant occupational health and safety measures that make employees aware of their work-related behavior, and that prevent evening work, longer work hours, and work in leisure time, include measures for recording work hours (Lott, 2020a), which help to increase workers’ awareness of their actual work hours and to curb long work hours. However, that is just one way to protect the work–life balance of workers and their families. The increasing intensification of work in the various labor market sectors (Kelly & Moen, 2020) reinforces “constant connectivity” (Wajcman & Rose, 2011) in a digitalized labor market, which results in long work hours, evening work, and work contact in leisure time. When the quantity of work does not match the time scheduled for it (Koltai & Schieman, 2015), workers feel overwhelmed by the workload and the lack of time to complete work tasks (Schieman, 2013). For these workers, work contact in leisure time, evening work, and long work hours are an ad hoc solution to this problem—with negative consequences for themselves (Guinchi et al., 2016) and, as the present study suggests, for their partners. Overload counteracts individual work–life balance measures as well as occupational health and safety and work–life balance policies. Therefore, workloads must be reduced at many workplaces. Adequate staffing, reliable substitute arrangements, and workloads that fit the contractual work hours, rather than vice versa, are key.

## Data Availability

The data that support the findings of this study were provided by the German Family Panel (pairfam; https://www.pairfam.de/en/data/data-access/). Restrictions apply to the availability of these data, which were used under license for the current study, and are therefore not publicly available. However, data are available from the authors upon reasonable request and with permission of the German Family Panel (pairfam).

## Appendix

### Results of the stratified analysis

Table 3

**Table 3:** Indirect effects of workers’ working time demands on workers’ and partners’ work–life balance satisfaction for part-time workers

Workers’ working time demands	Workers’ WLBS through workers’ WLC (H1)	Partners’ WLBS through workers’ WLC and partners’ WLC (H2a)	Partners’ WLBS through workers’ WLC and workers’ WLBS (H2b)
Answering work emails/phone calls in leisure time	− .11** (.03) [− .17; − .05]	− .02* (.01) [− .04; − .01]	− .00 (.01) [− .02; .01]
Evening work	− .09*** (.03) [− .16; − .03]	− .02* (.01) [− .02; − .01]	− .00 (.01) [− .02; .00]
Weekly work hours	− .15*** (.04) [− .22; − .08]	− .03** (.01) [− .05; − .01]	− .01 (.01) [− .02; .01]

*Note. N* = 338 dual-earner couples. WLC = work–life conflict; WLBS = work–life balance satisfaction; H = hypothesis. The table presents standardized coefficients, standard errors in parentheses, and lower and upper levels of 95% confidence intervals (bootstrapped) in brackets.

^*^ *p* < .05. ***p* < .01. ****p* < .001

Table 4

**Table 4:** Indirect effects of workers’ working time demands on workers’ and partners’ work–life balance satisfaction for full-time workers

Workers’ working time demands	Workers’ WLBS through workers’ WLC (H1)	Partners’ WLBS through workers’ WLC and partners’ WLC (H2a)	Partners’ WLBS through workers’ WLC and workers’ WLBS (H2b)
Answering work emails/phone calls in leisure time	− .10** (.02) [− .15; − .08]	− .01* (.01) [− .02; − .00]	− .01* (.00) [− .02; − .00]
Evening work	− .06*** (.02) [− .10; − .03]	− .01* (.00) [− .02; − .00]	− .00 (.00) [− .01; − .00]
Weekly work hours	− .12*** (.02) [− .15; − .06]	− .01 (.01) [− .03; .00]	− .01 (.01) [− .02; .00]

*Note. N* = 719 dual-earner couples. WLC = work–life conflict; WLBS = work–life balance satisfaction; H = hypothesis. The table presents standardized coefficients, standard errors in parentheses, and lower and upper levels of 95% confidence intervals (bootstrapped) in brackets.

^*^ *p* < .05, ***p* < .01, ****p* < .001

Figure 3

Figure 4
